# Identifying Prognostic SNPs in Clinical Cohorts: Complementing Univariate Analyses by Resampling and Multivariable Modeling

**DOI:** 10.1371/journal.pone.0155226

**Published:** 2016-05-09

**Authors:** Stefanie Hieke, Axel Benner, Richard F. Schlenk, Martin Schumacher, Lars Bullinger, Harald Binder

**Affiliations:** 1 Institute for Medical Biometry and Statistics, Medical Center- University Freiburg, Freiburg, Germany; 2 Freiburg Center for Data Analysis and Modeling, University Freiburg, Freiburg, Germany; 3 Division of Biostatistics, German Cancer Research Center, Heidelberg, Germany; 4 Department of Internal Medicine III, University Hospital of Ulm, Ulm, Germany; 5 Institute of Medical Biostatistics, Epidemiology and Informatics, University Medical Center Johannes Gutenberg University Mainz, Mainz, Germany; National Taiwan University, TAIWAN

## Abstract

Clinical cohorts with time-to-event endpoints are increasingly characterized by measurements of a number of single nucleotide polymorphisms that is by a magnitude larger than the number of measurements typically considered at the gene level. At the same time, the size of clinical cohorts often is still limited, calling for novel analysis strategies for identifying potentially prognostic SNPs that can help to better characterize disease processes. We propose such a strategy, drawing on univariate testing ideas from epidemiological case-controls studies on the one hand, and multivariable regression techniques as developed for gene expression data on the other hand. In particular, we focus on stable selection of a small set of SNPs and corresponding genes for subsequent validation. For univariate analysis, a permutation-based approach is proposed to test at the gene level. We use regularized multivariable regression models for considering all SNPs simultaneously and selecting a small set of potentially important prognostic SNPs. Stability is judged according to resampling inclusion frequencies for both the univariate and the multivariable approach. The overall strategy is illustrated with data from a cohort of acute myeloid leukemia patients and explored in a simulation study. The multivariable approach is seen to automatically focus on a smaller set of SNPs compared to the univariate approach, roughly in line with blocks of correlated SNPs. This more targeted extraction of SNPs results in more stable selection at the SNP as well as at the gene level. Thus, the multivariable regression approach with resampling provides a perspective in the proposed analysis strategy for SNP data in clinical cohorts highlighting what can be added by regularized regression techniques compared to univariate analyses.

## Introduction

In recent years, a multitude of molecular platforms have become available that provide a huge number of measurements for each individual, typically ten thousands to one million. While some of these platforms might be similar on a technical level, the type of research community, i.e. medical or epidemiological, in which the different measurement techniques are investigated depends on the specific molecular characteristics that are measured from such platforms. For example, gene expression microarrays or corresponding sequencing techniques have been used for some time in a clinical setting, where, e.g. the gene expression profile of a tumor might provide insight into the specific sub-entity, and allow for improved prognosis, if all phases of marker development are handled carefully [1, 2]. In contrast, single nucleotide polymorphism (SNP) microarrays have become a central component of large epidemiological case-control studies see, e.g. [3] for the impact of such data on nephrology research, and increasingly sequencing techniques are also used in this field, resulting in even more measurements. Already the microarrays allow to measure millions of potential genomic base pair changes, and thus might identify SNPs that characterize individuals with increased disease risk. While it might be feasible to reduce the number of SNPs [4], often all SNPs will have to be considered for statistical analysis.

The different medical/epidemiological communities might also be reflected in the corresponding statistical methods that typically are employed. There is a considerable number of multivariable techniques that incorporate all microarray measurements simultaneously, for developing a prognostic signature, see [5–7] for an overview and comparisons of some techniques. These signatures ideally should comprise only a small set of microarray features, i.e. genes. Correspondingly, many statistical approaches have been developed for providing variable selection in a high-dimensional multivariable modeling setting. Given the limited number of individuals in clinical cohorts, the resulting signatures will often be unstable [8], but might still provide reasonable prediction performance.

In contrast, epidemiological case-control studies will often be large enough to provide sufficient power for identifying risk-increasing SNPs, even if their effect is small. This is reflected in corresponding univariate statistical testing approaches with strict control of type I error rates, see [9] for an overview of strategies. While multivariable modeling techniques that provide variable selection, such as the lasso [10], have also been considered in the context of SNP data from large case-control studies, there has been only limited use so far [11, 12].

In the following, we consider clinical cohorts, where SNP microarray measurements are available for each patient at a baseline time. These are to be linked to a time-to-event endpoint. From a statistical point of view, the modeling challenge for such data is closer to gene expression analyses, as there is only a relatively small number of patients compared to a huge number of SNP covariates. However, lessons from large case-control study SNP measurements should not be ignored.

To obtain an overall analysis strategy, we will use a multivariable regression modeling approach for signature development to complement conventional univariate analyses, which are similar to techniques from epidemiological case-control studies. Specifically, componentwise likelihood-based boosting [13–15] is considered as a multivariable regression modeling approach, and adapted to the requirements of SNP data. For a general overview of boosting techniques see [16] and for componentwise boosting in particular [17].

An application with acute myeloid leukemia (AML) patients derived data, which is described in Section ‘Acute myeloid leukemia application’, is used for illustrating the overall strategy. The components of this strategy are described in the Section ‘Components of the analysis strategy’. The approaches are explored in a simulation study in the Section ‘Simulation study’. The results for the AML application are shown in the Section ‘Illustration for the AML application’, with a focus on the stability of selected lists of molecular entities, at the SNP and the gene level. We will specifically highlight the effect of SNP correlation structure on the results for the univariate test-based and the multivariable boosting approach. Some more general, concluding remarks are provided in the ‘Conclusion’.

## Acute myeloid leukemia application

Acute myeloid leukemia (AML), the most common acute leukemia in adults, represents a genetically heterogeneous disease. Currently, several clinically relevant genomic aberrations are known to distinguish AML at the molecular level [18, 19]. However, there exists a huge still unexplained heterogeneity within these identified molecular subtypes, specifically with respect to clinical endpoints, such as time-to-event.

Recently, we used high-resolution SNP analysis to delineate novel candidate disease genes in cases of AML [18]. To improve outcome prediction based on these genomic data sets, the outcome measure in AML needs to be carefully selected. An important outcome measure is relapse-free survival (RFS) [20]. Thus, we consider the endpoint RFS, i.e. relapse or death, whatever happens first, are the events of interest for illustrating the proposed univariate and multivariable analysis strategy. Data for patients alive (alive without relapse) were considered as censored. Overall, there is data from 308 patients, of which 154 had a relapse or died during their follow-up.

Beside age, white blood cell count (WBC), somatically acquired mutations in the nucleophosmin 1 (*NPM1*) and fms-related tyrosine kinase 3 (*FLT3-ITD*) genes as well as a cytogenetic risk group factor are known to be important predictors in AML cases that need to be taken into account as mandatory covariates for adjustment. We obtained 390443 SNPs with adequate quality control measures for 308 AML cases (n = 87 250k arrays; n = 221 6.0 SNP arrays). Each SNP is coded with values 0, 1 and 2 indicating the number of risk alleles for each patient and each SNP, see [21] for example.

## Components of the analysis strategy

### Model estimation

For a clinical cohort with a single time-to-event endpoint, such as the time to relapse or death, the observations are given by (*t*_*i*_, *δ*_*i*_, *x*_*i*_), *i* = 1, …, *n*, where *t*_*i*_ is the observed time for a patient which is the minimum of the survival time *T*_*i*_ and the censoring time *C*_*i*_. The event indicator *δ*_*i*_ = *I*(*T*_*i*_ ≤ *C*_*i*_) takes the value *δ*_*i*_ = 1 if its argument is true, i.e. if an event was observed, and 0 otherwise. The covariate vector *x*_*i*_ = (*x*_*i*, 1_, …, *x*_*i*, *r*_, *x*_*i*, *r*+1_…, *x*_*i*, *r*+*L*_)′, observed at time zero, comprises *r* established predictors of survival, such as clinical covariates that may be deemed mandatory, as well as one element for each of the *L* SNPs, which are coded as 0/1/2.

A common model for time-to-event data is the Cox proportional hazards model [22] given by

$$h\left(t|x_{i}\right)=h_{0}\left(t\right)\exp\left(\eta_{i}\right)=h_{0}\left(t\right)\exp\left(\sum_{j\in S}x_{ij}^{\prime }\beta_{j}\right).$$ (1)

The conditional hazard *h*(*t*|*x*_*i*_), i.e. the instantaneous risk of observing an event at time *t* conditioning on the covariate vector and survival up to time *t*, is modeled by an unspecified baseline hazard *h*_0_(*t*) and a linear predictor *η*_*i*_ with parameter vector *β* = (*β*_*j*_, *j* ∈ *S*)′, comprising the effects for SNP as well as for established (clinical) predictors.

The set *S* indicates the covariates to be considered for model fitting. In what will be called *univariate analysis* in the following, we consider one model per SNP, adjusted for the mandatory covariates, i.e. considering many models with corresponding sets

$$S_{uni,l}=\left\{1,\ldots ,r,l\right\}\;\;\;l=1,\ldots ,L.$$

For a multivariable analysis all SNPs are considered simultaneously, i.e.

$$S_{multi}=\left\{1,\ldots ,r,r+1,\ldots ,r+L\right\}.$$

Estimation of the parameter vector *β* is based on maximizing the partial log-likelihood

$$l\left(\beta \right)=\sum_{i=1}^{n}\delta_{i}\left(x_{i}^{\prime }\beta -\log\sum_{k=1}^{n}I\left(t_{i}\leq t_{k}\right)\exp\left(x_{k}^{\prime }\beta \right)\right)$$ (2)

**Fig 1:**
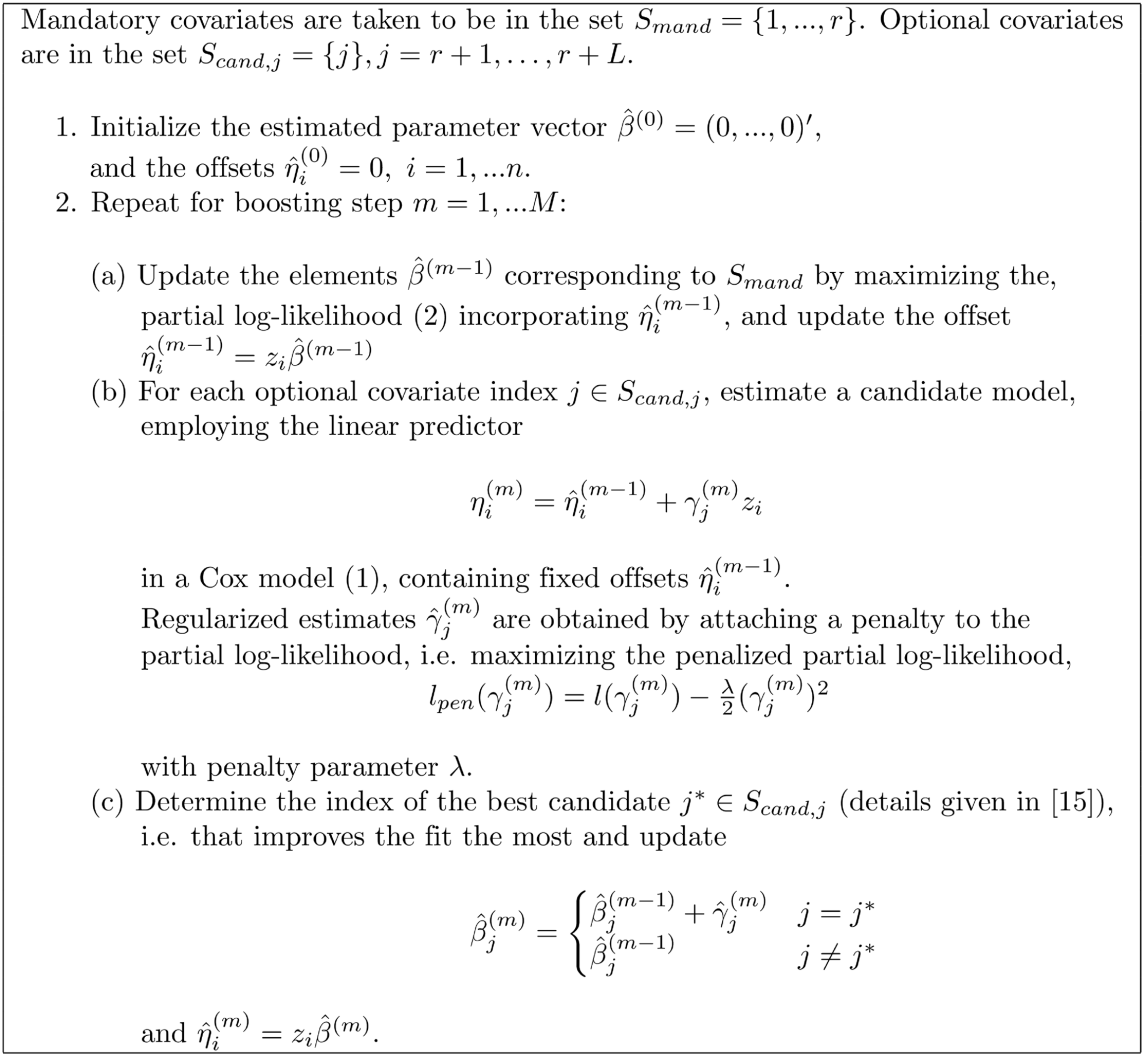
Componentwise likelihood-based boosting algorithm for time to event endpoint.

For the univariate analysis, standard maximum (partial) likelihood techniques are used. In the multivariable setting, straightforward maximization is no longer possible, but estimates can still be obtained by regularized techniques. In the following, we briefly describe a componentwise likelihood-based boosting approach, which shrinks many elements of the estimated parameter vector to zero, i.e. performs variable selection. A more formal description of the boosting algorithm is given in Fig 1.

Componentwise likelihood-based boosting [13, 15, 23] starts with an estimate where all elements are equal to zero, except those that correspond to mandatory covariates that need to be adjusted for. The effects *β*_*j*_, *j* = 1, …, *r*, of the latter are estimated by standard maximum (partial) likelihood techniques. The elements of the parameter vector *β* that do not correspond to such mandatory covariate are subsequently built up in a large number of boosting steps. In each step, only one of these elements is updated. The element to be updated is chosen to maximize a penalized (partial) log-likelihood. For determining these updates, one candidate model is fitted for each SNP, corresponding to a set *S*_*cand*, *j*_ = {*j*}, *j* = *r*+1, …, *r*+*L*, where the estimates for the mandatory covariates and the SNP parameter estimates from the previous boosting steps are included as a fixed offset. For obtaining small steps, a penalized likelihood is used to estimate the parameters of these candidate models, i.e. a penalty term is added to the partial log-likelihood. The penalty term comprises a penalty parameter *λ* that determines the size of the steps and the square of the candidate model parameter *γ*_*j*_. The best candidate model for the update is chosen such that the overall penalized partial likelihood improves the most. The parameter estimate ${\hat{\gamma }}_{j}$ from the best candidate model is used to update the corresponding element of the overall parameter vector $\hat{\beta }$. The elements of the parameter vector corresponding to mandatory covariates are re-estimated between each boosting step.

For determining the best update in a boosting step [23] suggested to consider unpenalized score statistics, in contrast to the penalized partial log-likelihood used for estimating the size of the updates. This avoids issues with standardization, which seems unnatural for 0/1/2 coded SNP covariates. Otherwise, selection of SNP covariates would depend on their variance, with strong preferential selection of SNPs with large variance. While such a preferential selection might even be wanted, it at least deviates from analysis of SNPs by univariate tests, as the latter do not such strongly depend on SNP variance.

Componentwise likelihood-based boosting is closely related to the popular lasso approach, where regularization is performed by directly attaching a penalty that comprises of the absolute values of the elements of the parameter vector *β* to the partial log-likelihood [24]. Similar to the lasso [25], componentwise boosting will typically assign a non-zero estimate only to one of the covariates from a group of correlated covariates. However, there is a difference to the lasso in presence of strong correlation structure. The lasso is more prone to non-monotone coefficient paths, i.e. the parameter estimate for a SNP might move towards zero and even become zero again as model complexity increases [26].

### Determining the number of SNPs to be selected

For the univariate as well as for the multivariable analysis, we consider the primary aim of identifying a (small) set of SNPs that can then be used for further wet lab steps, such as functional analysis.

For the multivariable approach using componentwise boosting, the main tuning parameter is the number of boosting steps which is typically selected according to optimize prediction performance by cross-validation. The penalty parameter *λ* is of minor importance as larger values can typically be offset by a larger number of boosting steps. The selected number of boosting steps limits the number of covariates with non-zero estimated coefficients, i.e. the number of selected covariates. The latter will typically be somewhat smaller than the number of boosting steps.

In the univariate analyses, a Wald test statistic and corresponding *p*-value can be calculated for each SNP. The SNPs can then be ranked according to the resulting p-values [27]. The top SNPs deemed to be important can either be determined with respect to family wise error rates (FWER) or false discovery rates (FDR) can be considered [28].

### Gene level analyses

While the fundamental models presented above are estimated at the SNP level, analysis at the gene level also is needed for biological interpretation. We briefly describe the task of linking SNPs to genes in the following, before taking the gene level perspective in the univariate and the multivariable part of the analysis strategy.

Depending on the microarray design, a single SNP can be located either within the annotated gene structure, i.e. the SNP falls into the exon region of 5’UTR (untranslated region), CDS (coding DNA sequence), 3’UTR or the intron region, or it is annotated as upstream (when the SNP is upstream of 5’ end of the gene) or downstream (when the SNP is downstream of the 3’ end of the gene) relative to the neighborhood genes, e.g. as specified on the Affimetrix Annotation file (downloaded in 2011), to be used in the following. This relationship annotation leads to some SNPs corresponding to different genes, i.e. a SNP is associated with one or more genes that is/are closest to it. Therefore, some SNPs have overlapping gene annotations but vice versa some genes can also be linked to several SNPs.

For the multivariable analysis, a gene might then be considered selected when a linked SNP is selected by the boosting approach. While a similar definition can be used for the univariate analyses, i.e. the genes linked to the top SNPs are considered as selected, in the following we also present a permutation approach to formally test for gene effects, based on the univariate SNP models.

After linking the SNPs to genes, each gene will typically be associated with several univariate SNP models and corresponding p-values. Techniques that summarize univariate tests at a gene level, have been developed for genetic case-control settings, and provide gene region-level summaries [29]. We specifically adapt the min P approach for the present time-to-event setting in the following. These resulting summaries integrate all single locus tests within a gene into a single test statistic that represents the gene level association. The empirical distribution of the test statistic for the gene level is obtained using permutation methods [30]. In permutation resampling, the phenotype, i.e. the case-control status in many epidemiological settings, or event time and status in the present clinical cohort setting, is randomly re-assigned without replacement to obtain a pseudo case-control status or time-to-event endpoint, respectively. Then, the same test statistic as in the original data is recomputed using the pseudo data. This procedure is repeated *B* times, to obtain a null distribution. Specifically, the min P test statistic considers the smallest *p*-value from trend tests for each SNP within each gene region in a case-control setting [29, 31]. The min P test combines the trend *p*-values over multiple loci into one value by recording the minimum *p*-value as a test statistic. This min P approach is widely used in epidemiological case-control studies [32–34].

For the present time-to-event clinical cohort setting, the *p*-values from the Cox proportional hazard models can be similarly aggregated per gene. For each SNP, a *p*-value *p*_*l*_ (*l* = *r*+1, …, *r*+*L*) is calculated using the Cox model. For each gene *k* = 1, …, *K*, the corresponding *p*-values *p*_(*k*, *j*)_ (*j* = 1, …, *M*_*k*_) of all *M*_*k*_ SNPs closest mapped to that gene are combined by obtaining their minimum resulting in a gene level test statistic

$$\Theta_{k}=\min_{1\leq j\leq M_{k}}p_{\left(k,j\right)}.$$ (3)

As indicated above, a null distribution is obtained via *B* permutation data sets, i.e. the event time and the status indicator is permuted *B* times to generate a set of *B* permutation samples. This relies on the assumption that the censoring distribution is independent of the SNPs [35].

Let $p_{\left(k,j\right)}^{\left(b\right)}$ be the *p*-value from the Cox model for the *j*th SNP assigned to gene *k* in the *b*th permutation sample. For the *b*th permutation, the permuted min P test statistic for gene *k* is then given by

$$\Theta_{k}^{\left(b\right)}=\min_{1\leq j\leq M_{k}}p_{\left(k,j\right)}^{\left(b\right)}.$$ (4)

The *B* values of this statistics serve as distribution under the null hypothesis. The permutation-based *p*-value for the gene region-level summary $p^{\Theta_{k}}$ for gene *k* is then computed as

$$p^{\Theta_{k}}=\frac{1}{B}\sum_{b=1}^{B}I\left(\Theta_{k}^{\left(b\right)}\leq \Theta_{k}\right),$$ (5)

i.e. the proportion of the possible permutations ${\left\{\Theta_{k}^{\left(b\right)}\right\}}_{b=1}^{B}$ which are equal to or smaller than the observed gene region-level summary Θ_*k*_ from the original data set. This procedure automatically takes into account the number of SNPs tested within a gene and their underlying linkage disequilibrium pattern [29].

### Judging stability by resampling

Selection stability was suggested as a criterion for judging the results of statistical approaches already three decades ago, but has recently received increased attention, fueled by high-dimensional molecular applications (for an overview see [36, 37]). The underlying idea is to perform model building (including variable selection) in several resampling data sets, and to consider for each covariate the proportion of data sets where it has been selected.

Naturally, selection stability cannot be the sole criterion but needs to be combined with methods that, e.g. maximize a likelihood or prediction performance with respect to the endpoint of interest. In this combination, selection stability has even been suggested for obtaining false discovery rates [38]. The latter approach considers rather stable selection, where at least some of the covariates are selected in more than half of the resampling data sets. As this might be problematic in a clinical cohort SNP application, due to low power, we only consider selection frequencies, without attempting to obtain false discovery rates.

Resampling can be performed with replacement, corresponding to the bootstrap, or without replacement, i.e. subsampling. Resampling with replacement introduces a bias that will affect approaches that depend on a tuning parameter [39]. Therefore, we use subsampling for generating data sets of size 0.632*n*.

Due to the array design of SNP microarrays, identified SNPs may frequently by located up- or downstream to a potentially causal SNP. Correspondingly, stable identification of a SNP does not necessarily mean that it is causal, but just that it is a stable representative.

For analyses at the gene level, each inclusion of a SNP that is mapped to one specific gene can be counted as inclusion of that gene, resulting in gene inclusion frequencies. For stability analysis, this can be expected to offset the effect of SNP correlation, where different SNPs might be selected from a set of correlated SNPs that are all mapped to the same gene, in different resampling data sets.

## Simulation study

### Design

To gain insight into the performance of the multivariable regression modeling and univariate test-based analyses, both approaches are compared in a simulation study. Following the simulation design in Binder [40], we consider a design with correlated blocks of SNPs within a gene. Within blocks, a SNP takes the same value as its left neighbor with probability 75%. At block boundaries this probability is 50%. The first SNP in each gene is generated independently from a binomial distribution for each allele with pre-specified minor allele frequency of 0.4. Data with a moderate sample size of *n* = 500 observations with non-informative censoring and two (clinical) covariates (binary and continuous) for adjustment as well as *p* = 300000 SNPs are simulated. Per gene, SNPs are arranged within 300 blocks with 10 SNPs (3000 SNPs per gene). Six SNPs are informative while the other SNPs carry no information. Five of the six informative SNPs are located on one gene where three and two of them, respectively, are within one block. A true parameter value of 0.5 is considered for the three SNPs located within the first block and a value of -0.5 for two SNPs located within the second block. At least one informative SNP with value -0.5 is put on a second gene. Survival times are computed from a Cox proportional hazard model with baseline hazard 0.1 as in [41], the censoring times are chosen to be uniformly distributed on [0, 10], i.e. 50% of the observations are censored. 100 repetitions are performed where the multivariable technique is compared to the univariate testing approach. The tuning parameter for the multivariable approach using componentwise likelihood-based boosting, e.g. the number of boosting steps, is selected by 10-fold cross-validation. The procedures are evaluated in terms of type I error (false positive rate) and power (true positive rate) considering SNPs with non-zero coefficients as estimated by componentwise likelihood-based boosting and SNPs with a FDR smaller or equal to a level of 0.05 by the univariate approach, respectively, as selected.

### Results

The simulated data sets are additionally used to investigate the computational demand of the multivariable and univariate approach, see also Binder [40]. In the following, we consider runs on a single compute core on an Intel Xeon E5 3.0 GHz processor. For *n* = 500 individuals and *p* = 10000 SNPs, the multivariable regression modeling using componentwise likelihood-based boosting with a fixed number of 500 boosting steps took about 408 seconds and the univariate approach took 35 seconds. Using 10-fold cross-validation for tuning parameter selection to optimize prediction performance, the multivariable approach took about 1489 seconds. However, the latter can be parallelized. Investigating parallelization on 10 CPUs the multivariable approach took about 306 seconds, i.e. a speed-up of factor 5 is obtained compared to the sequential execution. For all subsequent use of boosting, the number of boosting steps was selected via 10-fold cross-validation. For *p* = 100000 the multivariable approach took about 2900 seconds and the univariate approach about 534 seconds and about 8073 and 9509 seconds for *p* = 300000. For *n* = 800 individuals (*p* = 10000 SNPs) computations for the multivariable approach took about 760 seconds and for the univariate approach about 38 seconds as well as about 1090 and 44 seconds for *n* = 1000 individuals (*p* = 10000 SNPs).

Table 1 shows mean type I error and power of the multivariable regression modeling and univariate approach, respectively.

**Table 1:** Mean type I error and power of the different procedures with respect to non-informative and informative SNPs.

	mean Type I error		Power					
	cor	uncor	SNP2	SNP5	SNP9	SNP33	SNP37	SNP3023
univariate approach	0.36	≪ 0.05	0.98	1.00	0.95	0.73	0.79	0.25
multivariable CV	0.08	≪ 0.05	0.96	0.97	0.90	0.88	0.87	0.85
multivariable 500 steps	0.10	≪ 0.05	0.97	0.99	0.91	0.92	0.92	0.92

Approaches are univariate testing, multivariable modeling using componentwise likelihood-based boosting with number of boosting steps selected by 10-fold cross-validation, and alternatively, with a fixed number of 500 boosting steps. cor: 24 non-informative SNPs located within one of the three different blocks carrying six informative SNPs. uncor: 299970 non-informative SNPs not included within the blocks carrying the informative SNPs. SNP2, SNP5, SNP9, SNP33 and SNP37: informative SNPs located within the first and second block, respectively, on one selected gene and SNP3023: informative SNP located on another selected gene.

For the type I error, we distinguish between non-informative SNPs (24 SNPs) which are located within one of the three different blocks carrying six informative SNPs and the remaining non-informative SNPs (299970 SNPs) not included within these blocks. The first two columns show the mean type I error over 100 simulated data sets across the non-informative SNPs which are divided into these two groups. The remaining six columns contain the power for the individual informative SNPs. The univariate approach (first line) is outperformed by the multivariable regression modeling (second line) for the mean type I error of the 24 non-informative SNPs located within the three blocks carrying the informative SNPs. Both approaches perform similar for the mean type I error of the remaining non-informative SNPs. The type I error for the latter is well below 0.05, probably because both approaches preferentially select SNPs that are located within the three blocks, and thus less frequently select some of the remaining SNPs that are uncorrelated to the SNPs with true effects, given a limited number of SNPs that can be selected overall, as implied by a limited number of steps in boosting and the ranking in FDR calculation in the univariate approach. The multivariable approach performs somewhat better for the informative SNPs (SNP33 and SNP37) located within the second block and especially outperforms the univariate approach in terms of power for the separate informative SNP3023 which might be due to the fact that componentwise likelihood-based boosting can disentangle the SNP correlation structure.

In addition, results of mean type I error and power for the multivariable regression modeling with a fixed number of 500 boosting steps are given in the last row of Table 1. The findings illustrate that componentwise likelihood-based boosting runs into overfitting (see mean type I error of 0.10 for 24 non-informative SNPs) if the number of boosting steps is not selected via cross-validation to optimize prediction performance.

## Illustration for the AML application

**Fig 2:**
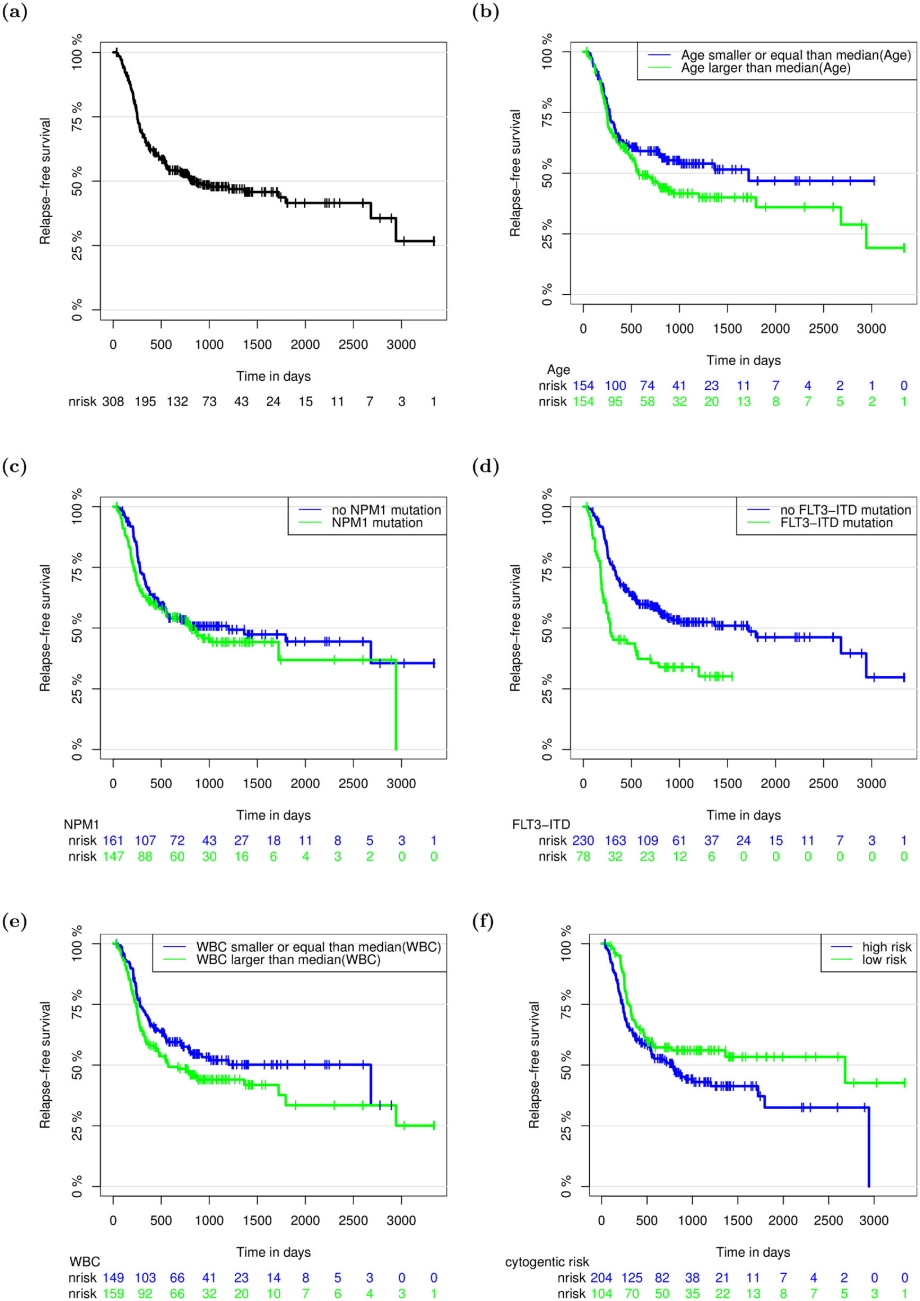
Relapse-free survival curves. The number of patients at risk at different time points are given below the graphs.

In the following we present results for the multivariable regression by componentwise likelihood-based boosting as part of an overall analysis strategy as well as for univariate analyses, and in particular compare the two lines of analysis for highlighting complementary results. Both approaches are adjusted for established clinical predictors referred to in the Acute myeloid leukemia application Section. For all predictors that were taken into account for adjustment in the multivariable and univariate analyses, respectively, their impact on RFS is shown in Fig 2.

Probabilities of RFS were calculated using the Kaplan-Meier method [42]. The median RFS was 863 days (Fig 2a). A reduced relapse-free survival was observed in patients aged older than 49 years (Fig 2b), in patients with somatically acquired mutations in NPM1 (Fig 2c) and FLT3-ITD (Fig 2d), respectively, in patients with WBC larger than 3.1 (Fig 2e) and in patients with a cytogentic risk factor (Fig 2f).

The multivariable approach is tuned, i.e. the number of boosting steps is selected, based on 10-fold cross-validation. For the univariate analyses, the SNPs are ranked according to the FDR. Specifically, the SNPs with FDR smaller or equal to a level of 0.05 are considered as selected in the original data and in resampling data sets, respectively.

### Multivariable regression by componentwise boosting

Four SNPs (rs5916016, rs256259, rs256215 and rs6541155), with minor allele frequency (MAF) of 0.4, 0.23, 0.34 and 0.24, respectively, are selected by componentwise likelihood-based boosting in the original data, i.e. they received non-zero estimates, when using 10-fold cross-validation for selecting the number of boosting steps. Concerning the array design, each of the four SNPs is annotated with one or more genes that is/are closest to it, yielding six mapped genes (*PRKX*, *NLGN4X*, *FSTL4*, *C5orf15*, *MARK1* and *C1orf115*). Five of the six genes correspond only to one of the selected SNPs (*PRKX* and *NLGN4X* are mapped to rs5916016, *C5orf15* corresponds to rs256215, *MARK1* and *C1orf115* are annotated with rs654115) and one gene (*FSTL4*) is mapped to two of the selected SNPs (rs256259 and rs256215). Therefore, the maximum number of selected SNPs from the original data corresponding to a gene equals two, i.e. these SNPs have overlapping gene annotations.

### Univariate approach

The univariate approach identifies three SNPs (rs256215 (MAF of 0.34) which corresponds to *FSTL4* and *C5orf15*, rs256225 (MAF of 0.31) is mapped to *C5orf15* and *FSTL4* and rs256259 (MAF of 0.23) is annotated with *FSTL4*) in the original data set while controlling the FDR at 0.05. To take relations between SNPs into account, the *p*-values from the univariate Cox models are summarized in the gene region-level summary, see Section ‘Gene level analyses’. This is, the min P test is investigated to combine the *p*-values computed over multiple SNP measurements *x*_*i*, *l*_ ∈ {0, 1, 2} (*i* = 1, …, *n*, *l* = *r* + 1, …, *r* + *L*) into one value representing the gene level association. After moving from the SNP level, i.e. 390443 univariate Cox models, to the gene level, there are still 17659 gene region-level summary tests.

As commonly done in genome-wide association studies, we consider the very conservative Bonferroni correction to take multiple hypothesis testing at the gene level into account. In addition, the FDR as a more sensible error control in presence of a large number of covariates for a small number of patients can be obtained by the approach of [28].

There is no permuted min P test statistic equal to or smaller than the observed min P test statistic from the original data for 5 genes (*FSTL4*, *C5orf15*, *OCIAD2*, *OCIAD1* and *SLC37A4*) in 10000 permutation samples which argues against the null hypothesis. However, the resolution of the permutation approach is potentially not sufficient enough. This highlights the limits of a permutation-based approach, which quickly becomes computationally unfeasible if more fine grained resolution is needed at small p-values. There is no further gene with an adjusted *p*-value or FDR smaller than or equal to 0.05. Even if a more lenient level such as 0.157 would be considered (roughly corresponding to selection based on AIC [43]) no further gene would be deemed significant.

### Univariate approach vs. multivariable regression

The findings of the multivariable and univariate approach, respectively, are evaluated at the SNP and the gene level. Table 2 (top left) shows the number of SNPs that have been selected by componentwise boosting in one or more resampling data sets. A median of eleven SNPs is selected based on cross-validation in the resampling data sets with range from 0 to 195. There is one resampling data set in which no SNP received a non-zero estimate, i.e. the optimal number of boosting steps is equal to zero. A SNP is counted as “IF > 0” if it received a non-zero estimate in one or more resampling data sets and included as well in “IF ≥ 10” if it is selected in ten or more resampling data sets. Only a small number of SNPs, compared to 390433 measured SNPs from the microarray, is selected in more than one resampling data set and many are selected only once. In addition, only few of these SNPs counted in “IF > 0” are included in “IF ≥ 10”. The “maximum IF” indicates the number of resampling data sets in which the most frequently selected SNP has been included. Correspondingly, the resampling inclusion frequencies for the SNPs are rather low compared to typical prognostic models. Note that this is far below the level of selection frequencies that would be needed for formally controlling false discovery rates according to [38]—partly due to the fact that the latter work can only provide a rather loose boundary, and partly due to a lack of power in a clinical cohort with rather limited size. We also consider the resampling inclusion frequencies at the gene level, counting a gene in a resampling data set if the gene is associated with an SNP selected in the corresponding resampling data set. Only a small number of genes, compared to 17659 genes mapped to 390433 SNPs, is counted in more than one resampling data set and also many are only selected once. However, moving from the SNP to the gene level results in a slightly larger number of genes which are selected in one or more resampling data sets and a tendency towards larger inclusion frequency for those genes compared to the SNP level. The larger inclusion frequency for genes that are selected in one or more resampling data sets, compared to SNPs, probably is due to the multiple annotations for each SNP as the chance rise that a gene is selected. However, Table 2 (bottom part) shows increased stability when considering maximum inclusion frequency compared to the SNP level (top part).

**Table 2:** Number of SNPs (top part) and number of genes (bottom part) with resampling inclusion frequencies (IF) larger than 0 and 10, and maximum IF in 100 resampling data sets as well as the overlap between the multivariable model and the univariate approach.

	multivariable model	overlap	univariate approach
	SNP level	SNP level	SNP level
IF > 0	395	58	218
IF ≥ 10	3	3	3
max	45	rs256215	28
	gene level	gene level	gene level
IF > 0	556	102	235
IF ≥ 10	4	2	2
max	72	*FSTL4*	34

The values are given for the multivariable and the univariate approach. Based on the FDR, for the latter the SNPs with *p*-values smaller or equal to 0.05 are considered.

We also evaluate the inclusion frequency for the selected SNPs in 100 resampling data sets based on the univariate approach. By applying the univariate test-based strategy in each resampling data set, a median of two SNPs with a range from 0 to 30 is identified. After multiple hypothesis testing correction, there are thirty-nine resampling data sets in which no SNP would be deemed to be significant by the univariate approach, i.e. there is no SNP with FDR smaller than or equal to the level of 0.05. The right part of Table 2 shows the resulting pattern of inclusion frequencies. The univariate approach results in a smaller number of SNPs with non-zero inclusion frequency and a smaller maximum inclusion frequency. This might indicate less stability for the univariate approach. Even at the gene level, the number of genes with non-zero inclusion frequency and the maximum inclusion frequency is smaller compared to componentwise likelihood-based boosting, still implying less stability for univariate selection. Table 2 also shows the overlap between the multivariable model and the univariate approach. For IF≥10, the multivariable model identifies all SNPs/genes that are selected by the univariate approach, plus two additional genes. The most frequently selected SNPs/genes agree between the respective models.

By moving from the SNP to the gene level, the gene region-level summary was investigated combining the *p*-values from the univariate Cox models into one value representing the gene level association to take relations between SNPs into account. The genes associated with the SNPs selected from the boosting method in the original data are compared to the genes with permutation-based *p*-values from the gene region-level summaries (Section Gene level analyses) < 0.0001. Two of the six genes associated with SNPs identified in the boosting model are within the genes with permutation based *p*-value < 0.0001. Both genes are also annotated with SNPs selected by the univariate approach in the original data. One of both genes is mapped to two of the selected SNPs and one corresponds to one selected SNP. Both genes annotated with SNPs identified by the univariate approach in the original data have a permutation-based *p*-value < 0.0001. As expected, analysis guided by gene-based approaches utilizing the min P test ameliorates the multiple testing problem and controls the overall gene wide false positive rate as the gene region-level summary reduces the number of tests compared to ignoring the gene structure by performing a separate test for each SNP.

For further comparison of the multivariable and univariate approach, we focus at one specific gene identified by both approaches in the original data. We consider gene *FSTL4* that is the most frequent counted gene and is associated with more than one SNP selected from the boosting approach and from the univariate test-based strategy in the original data. Gene *FSTL4* has 217 corresponding SNPs. Thus, we examine the inclusion frequency of all SNPs mapped to that gene using the univariate and the multivariable approach in the resampling data sets. Table 3 shows only those SNPs that were included at least in one resampling data set either from the univariate or multivariable approach. Table 3 indicates that, compared to the univariate test-based strategy, the multivariable approach automatically focuses on fewer SNPs and thus better highlights the potentially important SNPs.

**Table 3:** Inclusion frequencies (IF) for gene *FSTL4* and inclusion frequencies of the corresponding SNPs (ordered by position) included in at least one resampling data set from the multivariate and the univariate approach, respectively.

	multivariable (IF)		univariate (IF)		
Gene/SNP total (17659/390443)	gene level	SNP level	gene level	SNP level	*p*-value
***FSTL4* (217 mapped SNPs)**	72		34		<0.001
rs10479044		0		2	
rs256258		3		2	
rs256259*,^⋄^		25		23	
rs256209		1		2	
rs256215*,^⋄^		45		28	
rs256219		1		2	
rs256221		0		1	
rs256225^⋄^		20		21	
rs256228		1		0	
rs4958139		0		1	
rs2867328		0		1	

*p*-value: permutation-based *p*-value from the gene region-level summary for moving from the SNP to the gene level in the univariate approach (Section Gene level analyses).

*: Selected SNPs from the boosting model for the original data set.

^⋄^: Selected SNPs from the univariate Cox models with FDR smaller or equal to 0.05 in the original data.

**Fig 3:**
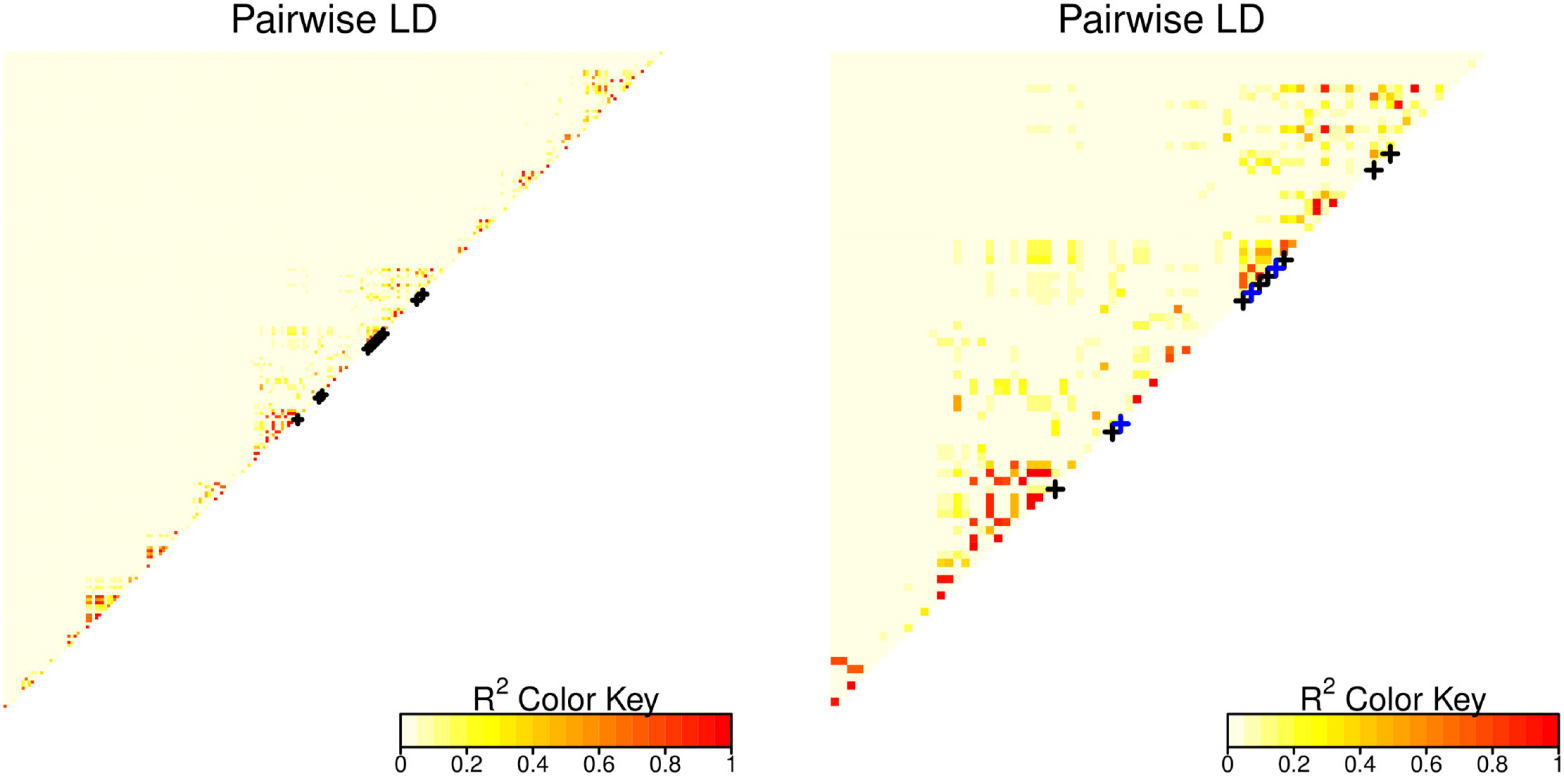
Linkage disequilibrium (*R*^2^) for the SNPs mapped to *FSTL4*. SNPs with non-zero inclusion frequencies are indicated by stars. The left panel shows all SNPs, the right panel only the region with the most frequently selected SNPs (IF > 20).

For investigating whether componentwise likelihood-based boosting can disentangle the SNP correlation structure in a reasonable way, we consider linkage disequilibrium (LD) plots for gene *FSTL4*. Fig 3 shows pairwise *R*^2^ for all 217 SNPs mapped to that gene (left panel) and more closely for the region with the most frequent selected SNPs (right panel). In the left panel, all SNPs with non-zero inclusion frequency are indicated by black stars (ordered by position, see Table 3). In the right panel, those SNPs with inclusion frequency larger than twenty for any of the two approaches are highlighted by blue stars. The blue highlighted SNPs have a *p*-value equal to or smaller than 0.05 from the univariate Cox models in the original data while controlling the FDR and two of them are also identified by componentwise likelihood-based boosting in the original data. The left panel already shows that several of the non-zero inclusion frequency SNPs belong to the same LD blocks.

Specifically, two of the most frequently selected SNPs seems to belong to the same LD block (right panel). As seen from Table 3, the boosting approach mostly picks one specific SNP (rs256215) from this block, while the univariate approach exhibits larger variability.

## Conclusion

A considerable number of statistical approaches has been developed for gene expression microarray data from clinical cohorts and for SNP microarray data from large epidemiological case-control studies. For the former, many techniques focus on developing prognostic or predictive signatures, while reliable identification of single risk-increasing SNPs is more important in the latter setting. We considered SNP microarray data from clinical cohorts with time-to-event endpoint as a setting that has received less attention so far, but is challenging due to the huge number of molecular measurements compared to a relatively small number of patients. Specifically, we applied componentwise likelihood-based boosting as a multivariable regression modeling technique that can select a small set of potentially important SNPs and indicated specific properties with respect to SNP data, such as strong correlation. As part of an overall analysis strategy, this was contrasted with an univariate test-based approach at the SNP level as well as at the gene level where a gene summary statistic with permutation-based *p*-values was suggested as a test-based gene-level analysis.

In an application to SNP data from AML patients, three SNPs were found to be significant by the univariate test-based approach fitted at the SNP level, while componentwise boosting selected four SNPs annotated with six genes. In contrast to the univariate approach, componentwise likelihood-based boosting was seen to avoid inclusion of SNPs from the same LD block within a gene. In this instance, automatic handling of correlation structure by componentwise boosting seems to provide reasonable results and might be the basis for increased power. This was also seen in a simulation study.

By moving from the SNP level to the gene level, the instability of univariate selection might be continued. Componentwise likelihood-based boosting was seen to benefit even more from the move to the gene level. Naturally, this requires reliable annotation for the SNP gene mapping. The fact that the genes identified by our analysis make biologically sense argues strongly in favor of a meaningful annotation in our analysis. For example, *FSTL4* (a member of the follistatin-like proteins; *FSTl1* instead of *FSTL4*) was identified as candidate gene affected by LOH in a murine cancer model [44]. This further highlights the strength of our data analysis approach as it revealed novel prognostic markers of high biological relevance, which argue against a false positivity of our findings.

In summary, we found componentwise likelihood-based boosting to provide an attractive approach for the analysis of clinical SNP data with time-to-event endpoint, when used as part of an overall analysis strategy. Stability as quantified by resampling techniques, was used to contrast the two approaches with respect to stable selection of SNPs. In addition, the boosting approach avoided inclusion of correlated SNPs as it disentangle the correlation structure of the SNPs to some extent. In contrast, the univariate approach was seen to select several SNPs from the same LD block.

While regularized regression techniques could so far not provide convincing improvement over univariate techniques for large case-control studies, the typically much smaller number of individuals in clinical cohorts might specifically favor multivariable approaches that were developed, e.g. for obtaining prognostic signatures from gene expression data. Therefore, it could be expected that regularized regression techniques, such as componentwise likelihood-based boosting, will be widely used for clinical cohort SNP data in the future.

Componentwise boosting was considered for contrasting a regularized regression technique and an univariate test-based approach using the common Cox model as basis for both. Alternative models, e.g. an accelerated failure time model, could have been used as basis for the univariate and multivariable approach. However, we chose the Cox model firstly, because of its widespread use, and secondly, it is the standard approach for AML survival data [45]. Consequently, we did not systematically check whether an accelerated failure time model might perform better. The promising results of componentwise boosting might motivate investigation of further multivariable techniques, such as lasso. More generally, regularized regression techniques could be expected to provide added value in terms of stability in an overall analysis strategy for SNP data in clinical cohorts highlighting what can be obtained by such multivariable regression approaches compared to univariate analyses.
